# A novel stepwise micro-TESE approach in non obstructive azoospermia

**DOI:** 10.1186/s12894-016-0138-6

**Published:** 2016-05-12

**Authors:** Giorgio Franco, Filomena Scarselli, Valentina Casciani, Cosimo De Nunzio, Donato Dente, Costantino Leonardo, Pier Francesco Greco, Alessia Greco, Maria Giulia Minasi, Ermanno Greco

**Affiliations:** Department Gynaecological-Obstetrical and Urological Sciences, Sapienza University, via del Policlinico n 155 cap, 00161 Rome, Italy; Centre for Reproductive Medicine, European Hospital, Rome, Italy; Department Urology, Sant’ Andrea Hospital, Sapienza University, Rome, Italy; Robotic Urology Department, Policlinico Abano Terme, Padova, Italy

**Keywords:** Micro-TESE, TESE, Azoospermia, Sperm retrieval, ICSI

## Abstract

**Background:**

The purpose of the study was to investigate whether micro-TESE can improve sperm retrieval rate (SRR) compared to conventional single TESE biopsy on the same testicle or to contralateral multiple TESE, by employing a novel stepwise micro-TESE approach in a population of poor prognosis patients with non-obstructive azoospermia (NOA).

**Methods:**

Sixty-four poor prognosis NOA men undergoing surgical testicular sperm retrieval for ICSI, from March 2007 to April 2013, were included in this study. Patients inclusion criteria were a) previous unsuccessful TESE, b) unfavorable histology (SCOS, MA, sclerahyalinosis), c) Klinefelter syndrome. We employed a stepwise micro-TESE consisting three-steps: 1) single conventional TESE biopsy; 2) micro-TESE on the same testis; 3) contralateral multiple TESE.

**Results:**

SRR was 28.1 % (18/64). Sperm was obtained in both the initial single conventional TESE and in the following micro-TESE. The positive or negative sperm retrieval was further confirmed by a contralateral multiple TESE, when performed. No significant pre-operative predictors of sperm retrieval, including patients’ age, previous negative TESE or serological markers (LH, FSH, inhibin B), were observed at univariate or multivariate analysis. Micro-TESE (step 2) did not improve sperm retrieval as compared to single TESE biopsy on the same testicle (step 1) or multiple contralateral TESE (step 3).

**Conclusions:**

Stepwise micro-TESE could represent an optimal approach for sperm retrieval in NOA men. In our view, it should be offered to NOA patients in order to gradually increase surgical invasiveness, when necessary. Stepwise micro-TESE might also reduce the costs, time and efforts involved in surgery.

## Background

Nonobstructive azoospermia (NOA) is a condition characterized by absence of sperm in the ejaculate due to impaired spermatogenesis. Nonobstructive azoospermia is reported in about 60 % of azoospermic patients and 15 % of all infertile men [1]. The histologic patterns associated with NOA include Sertoli cell-only syndrome (SCOS), maturation arrest (MA), hypospermatogenesis and sclera-hyalinosis. Nonobstructive azoospermic men could benefit from surgical sperm retrieval and assisted conception by intracytoplasmic sperm injection (ICSI). The goal of surgical sperm recovery is to retrieve an adequate number of sperm for ICSI.

Different approaches were used with the intention of increasing the chances of finding viable sperm in NOA patients and, at the same time, optimizing organ preservation [2–4]. The recent updated European Association of Urology (EAU) guidelines (2014) recommend testicular biopsy as the best procedure to provide a histological diagnosis and to find sperm. However, the same EAU guidelines are unclear as to the type of sperm retrieval procedure considered best and recommended for patients with NOA.

In 1999, Schlegel [5] reported a novel microsurgical method for testicular sperm extraction (microdissection TESE, or micro-TESE). The author introduced this technique with the aim of improving sperm recovery and reducing invasivity of TESE in patients with NOA.

During micro-TESE, with the use of an operating microscope, it is possible to identify and selectively extract larger seminiferous tubules which have higher probability of harboring spermatozoa.

A number of studies suggest that micro-TESE should become the standard in the management of men with NOA [5–9]. Micro-TESE appears to improve the frequency of successful sperm retrieval in NOA patients, despite the removal of dramatically less testicular tissue. Relevantly, the extraction of seminiferous tubules during micro-TESE does not compromise the subtunical blood vessels, therefore the testicular damage is reduced as compared to a standard TESE [5, 9–13]. Schlegel [5] reported an improvement of sperm retrieval from a rate of 45 % with conventional TESE to a rate of 63 % with micro-TESE. However, the literature reports acceptable recovery rates with different techniques including single TESE biopsy: 41.6–49.5 % [14, 15]; multiple conventional TESE: 52.5–56 % [16–18]; micro-TESE: 35–77 % [16, 19–24].

A conventional TESE biopsy with a single small incision is minimally invasive. A multiple conventional TESE with many superficial testicular incisions and tissue extractions is more invasive and can cause damage particularly to the testicular subtunical vessels. Micro-TESE could also entail some damage to testis: it requires an extended equatorial incision to expose the parenchyma. Some concern has recently been raised on the risk of hormonal impairment after micro-TESE due to testicular damage [12, 25, 26]. In addition, micro-TESE requires the need of specific surgical equipment and skills with increased costs and operative time.

Following our preliminary experience [27], in the present study we performed testicular sperm retrieval in NOA patients using a novel surgical approach consisting in steps of increasing invasiveness: we refer to this procedure as to “stepwise micro-TESE”. Our aim was to evaluate the performance of TESE versus micro-TESE by comparing the two methods on the same testicles in terms of sperm retrieval. We also provide the clinical outcome after ICSI.

## Methods

### Study population

In the present study, from March 2007 to April 2013, 343 NOA patients were referred for sperm retrieval at our fertility clinic. Azoospermia was diagnosed when the absence of sperm was observed in two semen samples after 600 g centrifugation and screening at 400x magnification using an inverted microscope, according to the World Health Organization guidelines [28]. Surgical sperm retrieval was performed either by means of single or multiple conventional TESE or by micro-TESE. All the procedures were performed by a single expert surgeon (GF). In March 2007, it was decided that all future micro-TESE would have been performed with the new stepwise micro-TESE approach, consisting in a three-steps biopsy, as described later. Indications for stepwise micro-TESE were previous unsuccessful TESE/micro-TESE, unfavorable histology (complete SCOS or MA) and/or Klinefelter syndrome (KS).

Sixty-four patients received stepwise micro-TESE. Information collected included a clinical history and examination: patient’s age was 35.4 ± 5.07; levels of serum FSH, luteinizing hormone (LH) and inhibin B were respectively 25.8 ± 12.45, 10.5 ± 6.57 and 19.7 ± 17.67. The etiology of azoospermia was defined for 38 patients whereas in the remaining 26 it was undefined: 25 were Klinefelter, 12 had a history of chriptorchidism and 1 with micro deletion of the AZFc. All patients were tested for Y-microdeletions. No patient with AZFa, AZFb, AZFab, AZFbc, AZFabc microdeletions were included in our stud group since it is our policy to discourage surgery (TESE or microTESE) in patients with these conditions due to the known no chance of success in sperm retrieval. Twenty-three out of the 64 patients with azoospermia had undergone previous unsuccessful sperm retrieval. Of the 64 patients, 2 (3.1 %) had a testicular volume >12 ml; 9 (14.1 %) had a volume between 6 and 12 ml, and 53 (82.82 %) had a severely reduced volume (<6 ml). No pre-operative hormonal treatment was planned in any patients; in particular, none of the KS men received testosterone replacement prior to surgery.

### Surgical technique

**Fig. 1:**
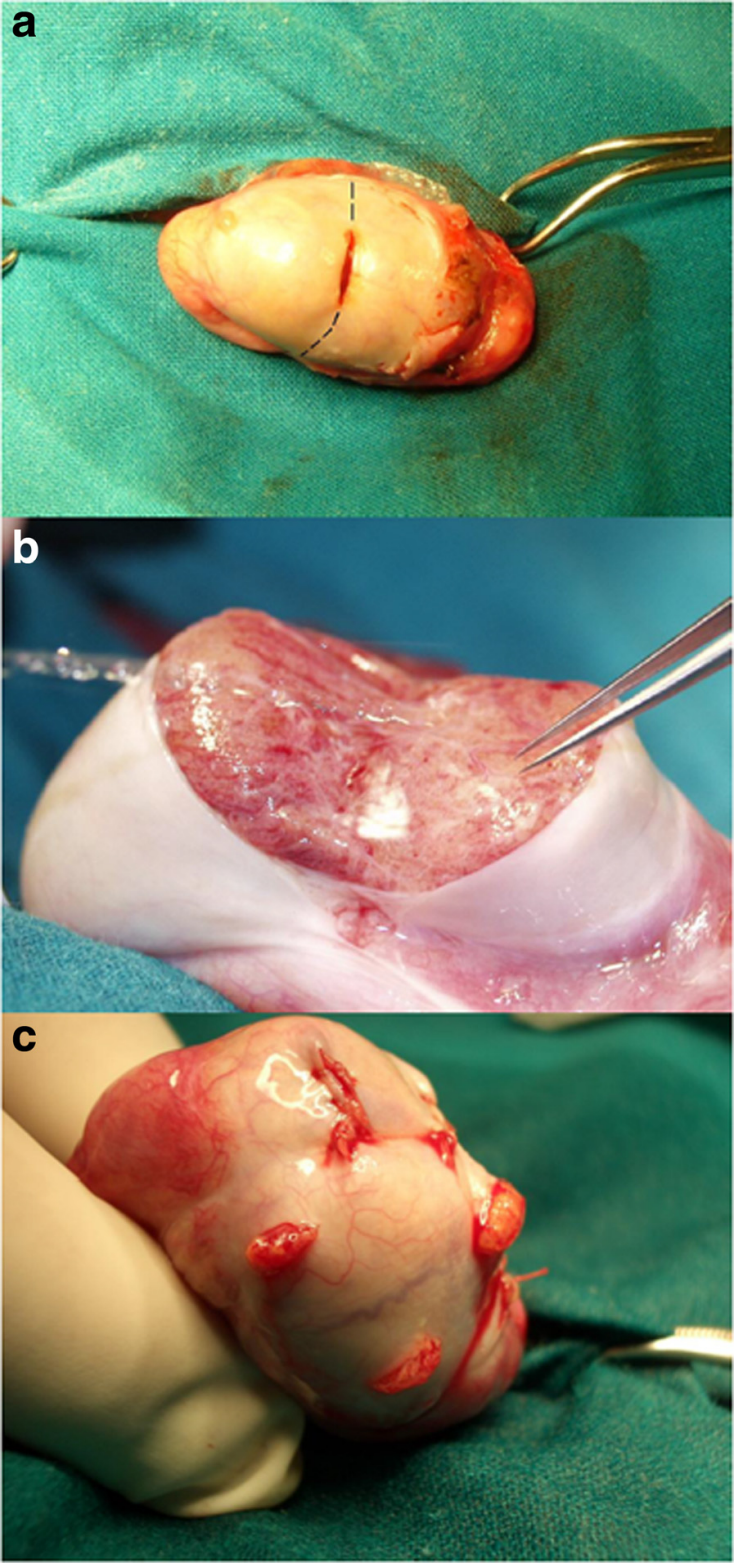
Stepwise microTESE; **a** Conventional single TESE: the dotted line indicates the extension of the original incision in order to perform micro-TESE; **b** micro-TESE; **c** contralateral conventional multiple TESE

The surgical procedure was performed under general anesthesia. After scrotal disinfection with iodopovidone and clorexidine digluconate, the spermatic cord and the scrotal skin were infiltrated with 8 ml of 7.5 mg/ml ropivacaine hydrochloride (Norepine, ASTA, Milan, Italy). The testicle on which the procedure was started was the one with larger volume or, in case no difference was evident between the two testicles, the procedure began on the right one, assuming that varicocele, if present, is on the left side. The scrotum was incised longitudinally for 2 cm on the median raphe and the testis was then delivered through the incision. The stepwise micro-TESE method consisted in three steps: for the initial TESE step, a small (5 mm) equatorial horizontal incision of the albuginea with extrusion of the testicular parenchima and scissors biopsy of approximately 5 × 2 × 3 mm (Fig. 1a). The second step consisted in a micro-TESE: under an operative microscope (10-24X magnification; Carl Zeiss, OPMI Surgical Microscope, Germany), an equatorial bilateral extension of the original incision was performed up to the hilum, with attention to preserving subtunical vessels. The testicle was then split open bluntly and tubules were retrieved with jewellers forceps from different sites of the two testicular sections (20 or more) aiming to locate and collect the larger ones with an increased chance of harboring spermatozoa (Fig. 1b); no attempt was made to retrieve tubules in the deep testicular parenchima [29]. At the end of the procedure, the albuginea incision was closed with a VICRYL 5/0 running suture. If no sperm was found so far, we proceeded with the third step: multiple conventional superficial biopsies (4–8 sampling) on the contralateral testicle (Fig. 1c). Each one of the multiple biopsies of the third step had identical procedure and sample size as described for the initial single TESE step. The surgical procedure was always performed by the same surgeon (GF) and the specimen processed by the same biological team.

For hystological examinations, a fragment of testicular parenchyma removed in the first TESE step was washed in buffered medium (Quinn’s Advantages Medium with HEPES, SAGE, Cooper Surgical, Pasadena, USA) with 2.5 % Human Serum Albumin (HSA, Albutein, Alpha Therapeutic Milan, Italy), fixed in Bouin’s solution (1 ml) and sent to the pathology laboratory. All histological examinations were performed by the same pathologist. The overall mean operating time was 1 h 30′ ± 30′. In our hands, mean operating time for micro-TESE is approximately 1 h 30′, while for single conventional TESE and for multiple TESE it is approximately 20′ and 45′ respectively.

The sequence of events during stepwise micro-TESE involves contemporarily the surgery room and the laboratory: starting from one testicle, the surgeon extracts the first tissue fragment (TESE) and places it in a Petri dish (containing 6–8 ml of buffered medium with HSA) which is immediately sent to the adjacent laboratory. Here, a biologist opens up the seminiferous tubules by mechanically dissecting the tissue with glass coverslides (wet preparation) as previously described [30]. The sperm search on the wet preparation is performed simultaneously by two biologists on two separate inverted microscopes (NIKON Eclipse S) at 400x magnification. After approximately 10–15 min, whether or not sperm are found in the wet preparation, the TESE sample is centrifuged (600G for 10 min). Part of the cell suspension is smeared and covered with mineral oil so that sperm search can continue on a more concentrated sample by one or more biologists simultaneously (for more severe cases even three or four biologists). If sperm is present, evaluation (motility, morphology) and quantification are provided. In parallel, after the initial tissue biopsy, the surgeon proceeds with micro-TESE. The microtubules extracted with micro-TESE are collected in a Petri dish and roughly shredded with coverslides as done for the initial TESE sample. If sperm are found in the two initial steps the contralateral biopies are avoided. Sperm search in the smeared samples can last even for 4–6 h, long after the surgery procedure is concluded. If sperm is not found on the day of surgery, the search continues on the next days for 1–2 h on the remaining cell suspensions. When sperm is found, it is either used on fresh ICSI cycles or cryopreserved for future use [30]. The samples retrieved during the different steps of the procedure were always treated and frozen separately.

### Statistical analysis

Statistical analysis was performed using the SPSS 12.0 software. Differences between groups of patients in medians for quantitative variables and differences in distributions for categorical variables were tested with the Kruskal-Wallis on way analysis of variance (ANOVA) and Chi-square test, respectively. Using multiple logistic regression with the enter method, variables evaluated in the univariate analysis were entered and investigated as predictors of sperm retrieval versus no sperm retrieval. The logistic regression analysis was carried out using data from patients for whom complete data were available. The variables considered for entry into the model included LH, FSH, Inhibin B, previous TESE (categorical variables). An alpha value of 5 % was considered as threshold for significance. Data are presented as median (range), mean ± standard deviation (SD). Odds ratios (OR) and 95 % confidence intervals (CI) were calculated for the parameters in each group, no sperm retrieval as reference group.

## Results

In 18 out of 64 patients we were able to retrieve testicular spermatozoa (sperm retrieval rate, SRR = 28.1 %). No significant differences in terms of sperm retrieval were observed between TESE and micro-TESE [18/64 patients for TESE versus 18/64 for micro-TESE (Chi square test, *p* = 1)]. In all cases in which we retrieved sperm with micro-TESE we had previously retrieved sperm with the initial single TESE, either immediately in the wet preparation or with a longer search after centrifugation. On the other hand, when no sperm was found in the first procedure, none was found in the second micro-TESE nor in the contralateral testicle (*N* = 46/64).

Of the 18 patients with positive sperm retrieval, 11 received only the two initial steps on the first testicle sparing the contralateral biopsy due to the fact that sperm was immediately found in the first testis. In the other 7 patients, sperm was not immediately found in the first testis with the two initial procedures, therefore we proceeded anyway on the contralateral testicle: all of them eventually had positive sperm retrieval in both testis.

The hystological evaluation performed on the specimens obtained from our 64 patients was: 37/64 patients (57.8 %) SCOS, 15/64 (23.4 %) MA, and 12/64 (18.8 %) sclera-hyalinosis. The histological diagnosis of positive sperm retrieval patients was: 10 MA, 1 sclera-hyalinosis and 7 SCOS. The histological diagnosis of negative sperm retrieval patients was: 5 MA, 11 had sclera-hyalinosis and 30 SCOS (Table 4). A significant (Chi square test, *p* = 0.001) higher sperm retrieval was obtained in patients with MA (10/15 = 67 % positive versus 5/15 = 33 % negative) when compared to patients with SCOS (7/37 = 18.9 % positive versus 30/37 = 81.1 % negative) and sclera-hyalinosis (1/12 = 0.8 % positive versus 11/12 = 99.2 % negative, Table 1). However, due to the small numbers of patients in each class, the statistical analysis has a reduced power.

**Table 1:** Histological diagnosis of patients with positive or negative sperm retrieval after stepwise micro-TESE. Significantly higher sperm retrieval was obtained in patients with MA when compared to patients with SCOS and sclera-hyalinosis

Histology	Positive sperm retrieval	Negative sperm retrieval	Chi square
MA (15 cases)	10/15 (67 %)	5/15 (33 %)	*P* = 0.001
SCO (37 cases)	7/37 (18.9 %)	30/37 (81.1 %)	
Sclera-hyalinosis (12 cases)	1/12 (0.8 %)	11/12 (99.2 %)	

The mean age of 18 men with positive sperm retrieval was 34.2 ± 7.16; mean FSH level was 25.9 ± 15.10, mean LH level was 12.5 ± 9.40 and mean inhibin B level was 14.9 ± 5.63. On the other hand, in the 46 with negative sperm retrieval, mean age was 35.9 ± 3.94. Average levels of FSH, LH and inhibin B were 25.7 ± 11.60, 9.7 ± 4.88 and 20.0 ± 18.86, respectively. No significant differences for each of these variables were observed between patients with positive and negative sperm retrieval (Table 2). Twenty-three patients had previously undergone a TESE elsewhere, and for 6 of them stepwise micro-TESE resulted in a positive sperm retrieval (26 %). On the other hand, of the 41 patients who had not previously undergone a TESE elsewhere, 12 had positive sperm retrieval with step-wise micro-TESE (29 %; Chi square test, NS; Table 3). With a multiple logistic regression the predictive value of serological markers (FSH, LH and inhibin B) and of a previous unsuccessful TESE was investigated (Table 4). No significant pre-operative predictors of sperm retrieval, including previous TESE or serological markers, were observed at univariate or multivariate analysis.

**Table 2:** Mean Age and preoperative variables (FSH, LH and inhibin B) in patients with positive or negative sperm retrieval after stepwise micro-TESE: none of these variables was predictive of sperm retrieval

	Positive	Negative	*p*
N	18	46	
Mean age	34.2 ± 7.16	35.9 ± 3.94	0.191
FSH	25.9 ± 15.10	25.7 ± 11.60	0.525
LH	12.5 ± 9.40	9.7 ± 4.88	0.586
Inhibin B	14.9 ± 5.63	20.0 ± 18.86	0.887

**Table 3:** Sperm retrieval rate was similar in the groups of patients who underwent or not a previous unsuccesful TESE

	Previous TESE	No previous TESE	Chi square
Positive sperm retrieval	6/23 (26 %)	12/41 (29 %)	*P* = 0.552

**Table 4:** Multiple logistic regression with the enter method. Variables evaluated in the univariate analyses were entered and investigated as predictors of sperm retrieval versus no sperm retrieval

	OR	95.0 % C.I.		*P*
FSH	0.979	0.863	1.112	0.749
LH	1.035	0.829	1.293	0.760
Inhibin B	0.944	0.787	1.132	0.532
Previous TESE	0.257	0.014	4.584	0.356

Stepwise micro-TESE was always optimally tolerated by patients with minimal post-operative pain and no major complications. Fifteen out of the 18 patients with positive sperm retrieval had sperm cryopreservation whereas 3 of them underwent an ICSI cycle with fresh sperm. Our results in terms of fertilization and live birth rate are comparable with those in the literature. Overall, 2 patients (1 KS with AZFc deletion and 1 SCOS) had unsuccessful fertilization both with cryopreserved sperm. Four patients had embryo transfer with negative beta-HCG (1 MA and 1 SCOS with cryopreserved sperm; 2 MA with fresh sperm). One SCOS patient had positive beta-HCG with cryopreserved sperm but no heartbeat was observed. One MA patient underwent an ICSI cycle with fresh sperm resulting in abortion at 8 weeks of gestation. Finally 3 ICSI cycles (all MA with cryopreserved sperm) ended with 4 babies delivered: 1 twin (1 female and 1 male) and 2 singleton (2 males) pregnancies (live-birth rate 27.2 % = 3/11 ICSI cycles).

## Discussion

In this study, a novel stepwise micro-TESE technique was performed on 64 patients with severe NOA undergoing testicular sperm extraction. First, a single TESE sample was taken from one testicle and, after this, a micro-TESE was performed extending the same testicular incision. The third step consisted in contralateral conventional multiple biopsies in case of negative sperm retrieval on the first testis. The rationale behind our study design was to explore the possibility of a gradual sperm retrieval approach aiming to minimize invasiveness, and to compare the efficiency of micro-TESE with conventional TESE. In our fertility center, surgery proceeds in parallel with sperm search in the laboratory and we are used to move to the contralateral testis in all cases of negative sperm retrieval, in order to maximize the chance of success. The contralateral multiple TESE served also as an additional control that micro-TESE was correctly performed in the first testis. Unexpectedly, no differences were seen in SRR among the initial TESE, the following micro-TESE and the final contralateral multiple TESE.

Conventional multiple TESE consists in random incisions which may result in atrophy and devascularization of the surrounding testicular tissue. This effect, together with the intratesticular bleeding and scar formation, can easily damage the spermatogenetic pathway and hormone production [8]. Micro-TESE with microtubules extraction from superficial sites of the section is less invasive than conventional multiple TESE [5]. This method, thanks to the optical magnification, allows a sparing of subtunical albuginea and intratesticular vessels with a minimal excision of testicular parenchyma. In fact, single seminiferous tubules can be collected without impairing the surrounding tissues. In the literature, there are many reports indicating micro-TESE as a more efficient method to retrieve spermatozoa with reasonable scarce invasiveness when compared to multiple biopsies. Tsujimura and collegues [13] reported a SRR of 45 % obtained with salvage micro-TESE performed after previous failed conventional TESE. Overall, the reported rates of successful sperm retrieval with micro-TESE varies between 47 and 66 % [7, 8, 12, 31, 32]. However, in our view, it is reasonable to believe that many of these successful micro-TESE cases might have benefited from a less invasive approach of sperm retrieval. In these studies, micro-TESE was apparently offered to all NOA patients, even those with good prognosis. For instance, the operated population often included NOA patients with the histologic pattern of hypospermatogenesis, although it is well known that SRR in this situation is very high with any sperm retrieval technique [33].

We operate in a private fertility center where micro-TESE, due to its higher costs (approximately 50 % higher than for conventional TESE) is offered only to NOA patients with severe clinical diagnosis and prognosis (complete SCOS or MA, KS, previous unsuccessful TESE). This might be an explanation why our overall SRR of 28.1 % (18/64) is low when compared to those reported in the literature. However, it should be noticed that in other reports, when looking only to NOA subpopulations with severe prognosis, a similarly low SRR was reported, as described in a detailed review from Ghalayini et al. [24].

In contrast with what expected, our micro-dissection step did not improve the chance of finding sperm: the SRR obtained with micro-TESE or with the initial single conventional TESE coincided. Moreover, the multiple TESE performed on the contralateral testicle always confirmed the outcome obtained on the first testicle, either successful or not.

A possible reason for our micro-TESE not improving SRR could be that our tubule collection method during micro-TESE remained superficial. Ramasamy and colleagues [29] have recently reported their technique of micro-TESE starting with a superficial extraction of tubules followed by a deeper and more extensive search below the superficial section. This second step improved the SRR by 18.4 % [29]. It is reasonable to assume that by extending our tubules collection to the deeper part of the testis, our SRR could be improved as well. However, one must take into account that an extensive procedure might entail additional damage and significantly prolong surgical time [34].

In another study from Marconi and colleagues [32], four surgical methods were compared, namely unifocal conventional TESE, unifocal micro-TESE, trifocal conventional TESE and trifocal conventional TESE plus micro-TESE. Consistently with our results, no difference was observed between micro-TESE and trifocal conventional TESE. Only the combination of trifocal conventional TESE plus unifocal micro-TESE significantly increased the SRR when compared to unifocal conventional TESE.

A surgical approach similar to ours has been applied by Turunc and colleagues [31]. The authors performed exclusively micro-TESE in a subgroup of severe NOA patients with testicular atrophy, obtaining a SRR of approximately 20 %. Another larger group of patients with a less severe prognosis, underwent firstly a trifocal conventional TESE. Only when no sperm was found, micro-TESE was performed by joining the three original incisions, therefore increasing the SRR from about 34 % up to 51 %. Anyway, it has to be pointed out that the majority of successes were obtained with the initial conventional TESE. These data support our belief that micro-TESE should not be offered indiscriminately to all NOA patients but a gradual approach should be warranted.

A large proportion of our population was represented by men with KS (25/64 = 39.1 %). In our view this condition is one of the most severe forms of NOA [30]. Of these 25 patients, 6 had positive sperm retrieval (24 %). Theoretically, micro-TESE should be an ideal approach for men with KS who are characterized by small testes, extensive sclera-hyalinosis and scattered areas of remaining tubules. In this situation, the magnification system used during micro-TESE helps in identifying the tubules among the sclerotic tissue. The SRR of KS man in the present series resulted considerably low when compared to other published studies, including one from our group (ref. 30). A possible explanation is that, in the present study group, the mean age of KS patients (35.5 ± 5.18) was substantially higher than that reported in other studies [30, 35, 36]. It is in fact well known how sclera-hyalinosis increases and spermatogenesis declines with age in KS patients.

The histology in our patient’s population was represented by SCOS (57.8 %), MA (23.4 %) and sclera-hyalinosis (18.8 %). The condition of hypospermatogenis, a less severe form of NOA, was not represented among our 64 patients. Interestingly, we observed a significantly higher SRR in patients with MA (Table 1) when compared to the other histological diagnosis, although our sample size is limited to obtain definitive conclusions. On the contrary, sclera-hyalinosis appeared to be related to a scarce chance of retrieving sperm. The literature is still controversial in terms of the prognostic value of testicular histology. Some authors report a higher success rate of micro-TESE in case of SCOS [21, 29]. This could be reasonably due to an incomplete SCOS, a condition in which larger microtubules with spermatogenesis can be easily identified with the optical magnification among those with only Sertoli cells. The histological diagnosis of our patients population can explain our low SRR in the present series: most of our patients had and unfavorable histology of SCOS (57.8 %) and sclera-hyalinosis (18.8 %); only a smaller proportion had MA (23.4 %) and nearly all of them had positive SRR (10/15).

In our experience, no significant pre-operative predictors of sperm retrieval, including previous TESE or serological markers, were observed at univariate or multivariate analysis (Tables 2, 3 and 4). Particularly, in our study, levels of FSH, LH, inhibin B were not predictive of sperm retrieval, consistently to the results reported elsewhere [37, 38]. In contrast, other groups reported a positive predictive power of some preoperative variables, such as FSH (39; 17) and inhibin B [39, 40]. Furthermore, in the subgroup of patients who had undergone a previous negative TESE elsewhere (*N* = 23, SRR = 26 %), the SRR did not differ from the subgroup of patients who had not received a previous negative TESE (*N* = 41, SRR = 29 %, Table 3).

## Conclusions

Our study indicate that 1) in patients with poor prognosis NOA even micro-TESE did not improve the chance of retrieving sperm; 2) in all patients with successful sperm retrieval, the initial, less invasive single conventional biopsy would have been enough to obtain sperm; 3) micro-TESE was always optimally tolerated by patients and left minimal if no scars; 4) due to the priority that should be always given to organ preservation, we believe that the gradual approach of stepwise micro-TESE could be ideal for testicular sperm retrieval in all NOA men. In this way costs, time and efforts involved in the surgery procedure would be drastically reduced.

### Ethics approval and consent to participate

The study was approved by the Institutional Ethics Committee of European Hospital. All patients were informed on the aspects of the surgical procedure and signed a written informed consent.

### Consent for publication

Not applicable.

### Availability of data and materials

All the data supporting our findings is contained within the manuscript.

## Abbreviations

AZF: azoospermia factor
beta-HCG: beta human chorionic gonadotropin
EAU: European Association of Urology
FSH: follicle stimulation hormone
HSA: Human Serum Albumin
ICSI: intracytoplasmic sperm injection
KS: Klinefelter syndrome
LH: luteinizing hormone
MA: maturation arrest
micro-TESE: microdissection testicular sperm extraction
NOA: non-obstructive azoospermia
SCOS: Sertoli cell-only syndrome
SRR: sperm retrieval rate
TESE: testicular sperm extraction
